# Multidisciplinary treatment of giant thymoma, paving the way to complete surgical resection: a case report

**DOI:** 10.1186/s40792-024-01970-2

**Published:** 2024-07-12

**Authors:** Ayaka Makita, Shota Nakamura, Tomohiro Setogawa, Yoshito Imamura, Shoji Okado, Yuji Nomata, Hiroki Watanabe, Yuta Kawasumi, Yuka Kadomatsu, Harushi Ueno, Taketo Kato, Tetsuya Mizuno, Toyofumi Fengshi Chen-Yoshikawa

**Affiliations:** Department of Thoracic Surgery, Nagoya University Graduate School of Medicine, 65 Tsurumai-Cho, Showa-Ku, Nagoya, 466-8550 Japan

**Keywords:** Thymic malignancy, Thymoma, Multidisciplinary treatment, Induction chemotherapy, Thymic epithelial tumor

## Abstract

**Background:**

A multidisciplinary treatment approach is recommended for patients with extensive, advanced, or recurrent thymomas. However, detailed treatment strategies, such as chemotherapy regimens and optimal surgical procedures, are still under debate.

**Case presentation:**

We report a case of gigantic locally advanced thymoma. A 70-year-old male was referred to our hospital following the detection of abnormal chest shadows. Chest X-ray and computed tomography (CT) scans revealed a 21-cm mass in the anterior mediastinum, encircling the pulmonary hilum and extending into the left thoracic cavity. PET/CT showed increased ^18^F-fluorodeoxyglucose uptake at the tumor site. Based on a trans-percutaneous CT-guided needle biopsy, the tumor was diagnosed as a Type B2 thymoma at the clinical IIIA stage. The patient underwent four cycles of preoperative induction chemotherapy, including cisplatin, doxorubicin, and methylprednisolone (CAMP), resulting in a partial response; the tumor shrank to 12 cm and FDG uptake decreased. Considering the patient’s age and comorbidities, we performed total thymectomy, along with partial resections of the parietal, mediastinal and visceral pleura, pericardium, and left upper lobectomy. This approach achieved complete histological resection, mitigating the risk of recurrence. Pathological analysis confirmed a thymoma, ypT3 (lung) N0M0 stage IIIA, with no malignancy in the pericardial or pleural effusions. No recurrence was detected 9 months post-surgery.

**Conclusions:**

We report a case of giant thymoma successfully treated with multidisciplinary strategy. Surgical treatment alone may not have achieved complete resection, but after inducing significant tumor shrinkage with preoperative CAMP therapy, we were able to achieve complete resection. This treatment strategy may be effective in large thymoma cases.

**Supplementary Information:**

The online version contains supplementary material available at 10.1186/s40792-024-01970-2.

## Background

A multidisciplinary treatment approach is recommended for patients with extensive, advanced, or recurrent thymomas [1]. However, detailed treatment strategies, such as chemotherapy regimens and optimal surgical procedures, are still under debate. We report a case of gigantic locally advanced thymoma. Successful complete resection of the thymoma was achieved by combining induction chemotherapy and surgical resection.

## Case presentation

**Fig. 1:**
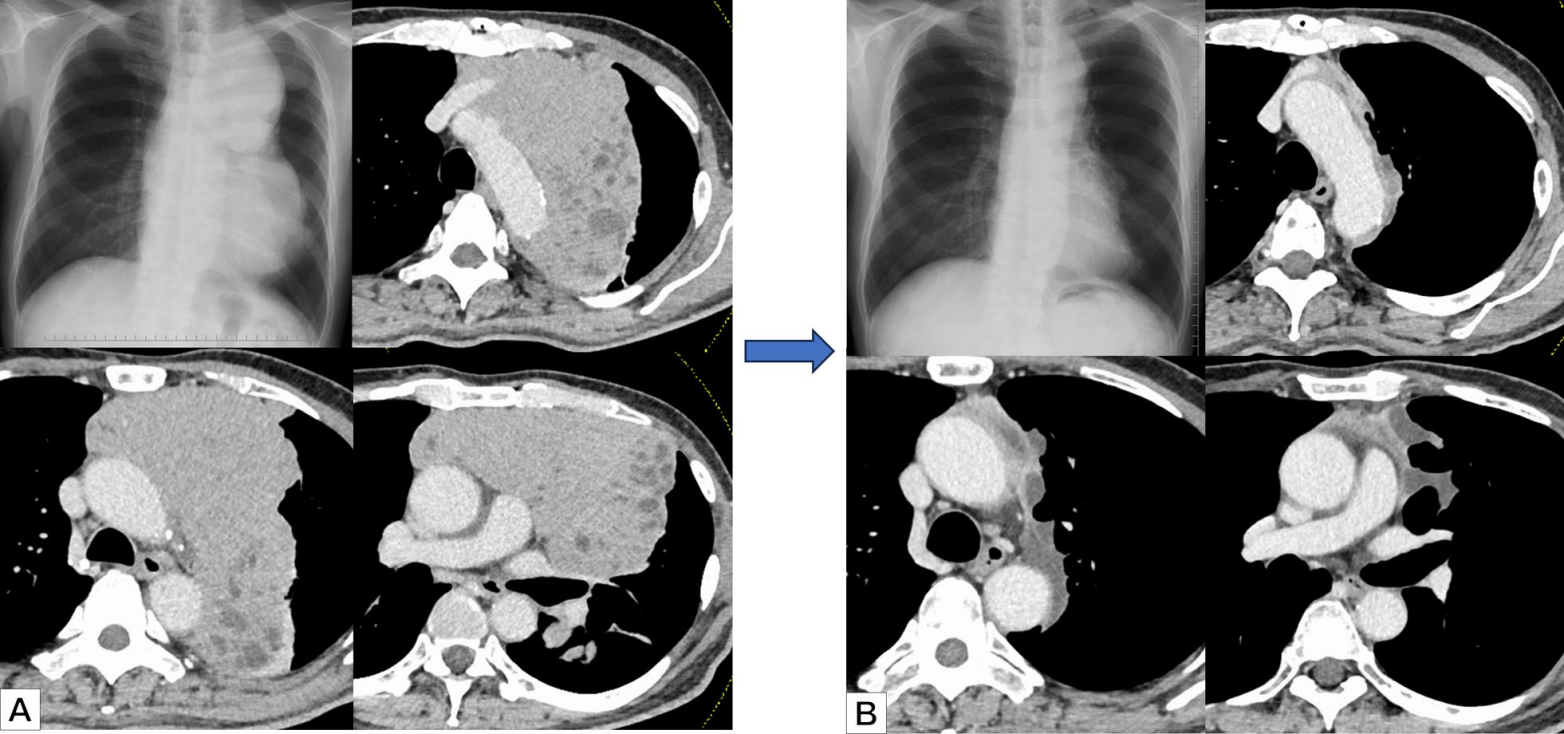
**A** Before any treatment, chest X-ray and computed tomography images revealed a 21 cm horseshoe-shaped mass in the anterior mediastinum, encircling the pulmonary hilum and extending into the left thoracic cavity. **B** After four cycles of preoperative induction chemotherapy with cisplatin, doxorubicin, and methylprednisolone (CAMP), chest X-ray and computed tomography images revealed tumor shrinkage to 12 cm and a partial response

**Fig. 2:**
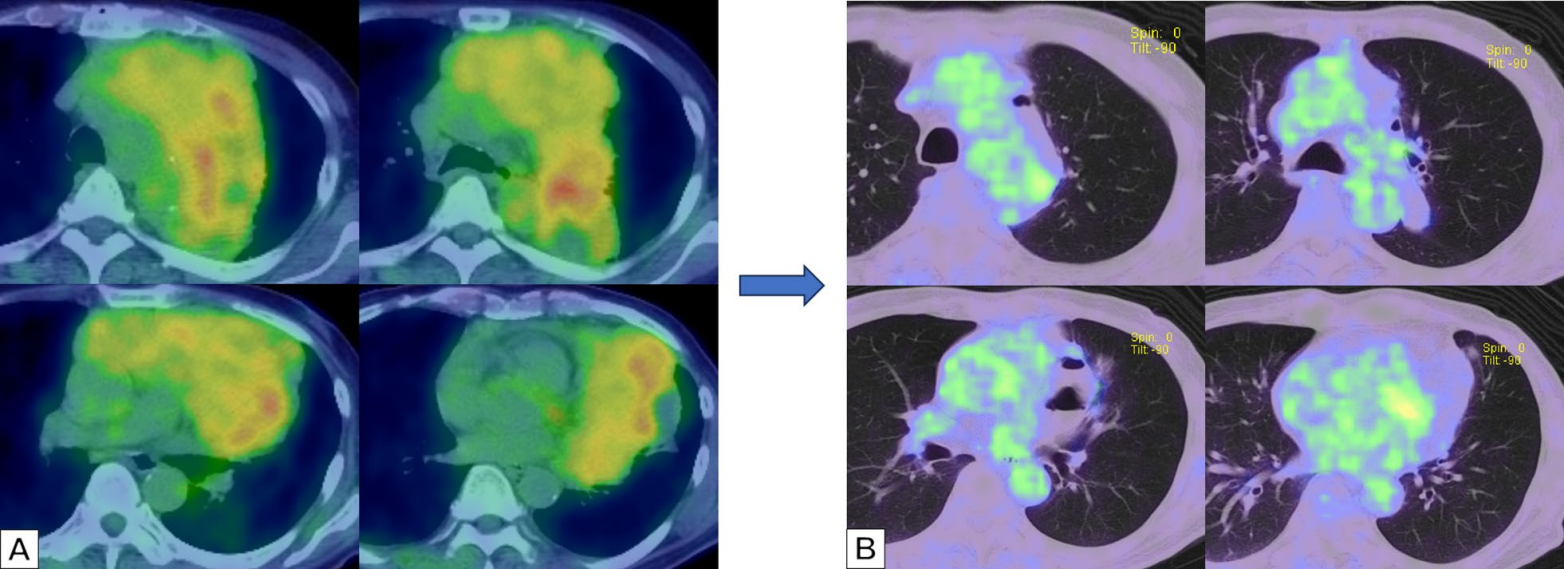
**A** Positron emission tomography/computed tomography image taken before treatment revealed increased ^18^F-fluorodeoxyglucose uptake (standard uptake max value 4.07) at the tumor site. **B** Positron emission tomography/computed tomography image taken after four cycles of preoperative induction chemotherapy with cisplatin, doxorubicin, and methylprednisolone (CAMP). Tumor ^18^F-fluorodeoxyglucose uptake decreased (standard uptake max value to 2.75)

A 70-year-old male was referred to our hospital following the detection of abnormal chest shadows. Chest X-ray and computed tomography (CT) scans revealed a 21-cm mass in the anterior mediastinum, encircling the pulmonary hilum and extending into the left thoracic cavity (Fig. 1A). PET/CT showed increased ^18^F-fluorodeoxyglucose uptake at the tumor site (Fig. 2A). There were no symptoms suggestive of myasthenia gravis. The preoperative antiacetylcholine receptor antibody level was < 0.2 nmol/L. Based on a trans-percutaneous CT-guided needle biopsy, the tumor was pathologically diagnosed as a Type B2 thymoma at the clinical IIIA stage (cT3(lung)N0M0). The patient underwent four cycles of preoperative induction chemotherapy, including cisplatin, doxorubicin, and methylprednisolone (CAMP), resulting in a partial response; the tumor shrank to 12 cm (Fig. 1B) and FDG uptake decreased (Fig. 2B). The chemotherapy regimen comprised cisplatin (20 mg/m^2^ per day, continuous infusion on days 1–4), doxorubicin (40 mg/m^2^ intravenously on day 1), and methylprednisolone (1000 mg/day intravenously on days 1–4 and 500 mg/day intravenously on days 5 and 6) (CAMP regimen). Treatment cycles were repeated every 21–28 days. Prophylactic granulocyte colony-stimulating factor was not used routinely. In total, four courses of chemotherapy were administered preoperatively. The 1st day of the first course was initiated 98 days before the surgery, and the surgery was performed 42 days after the 1st day of the fourth course. Chemotherapy induced Grade 2 adverse events, including supraventricular tachycardia, hyponatremia, hypomagnesemia, and anorexia.

**Fig. 3:**
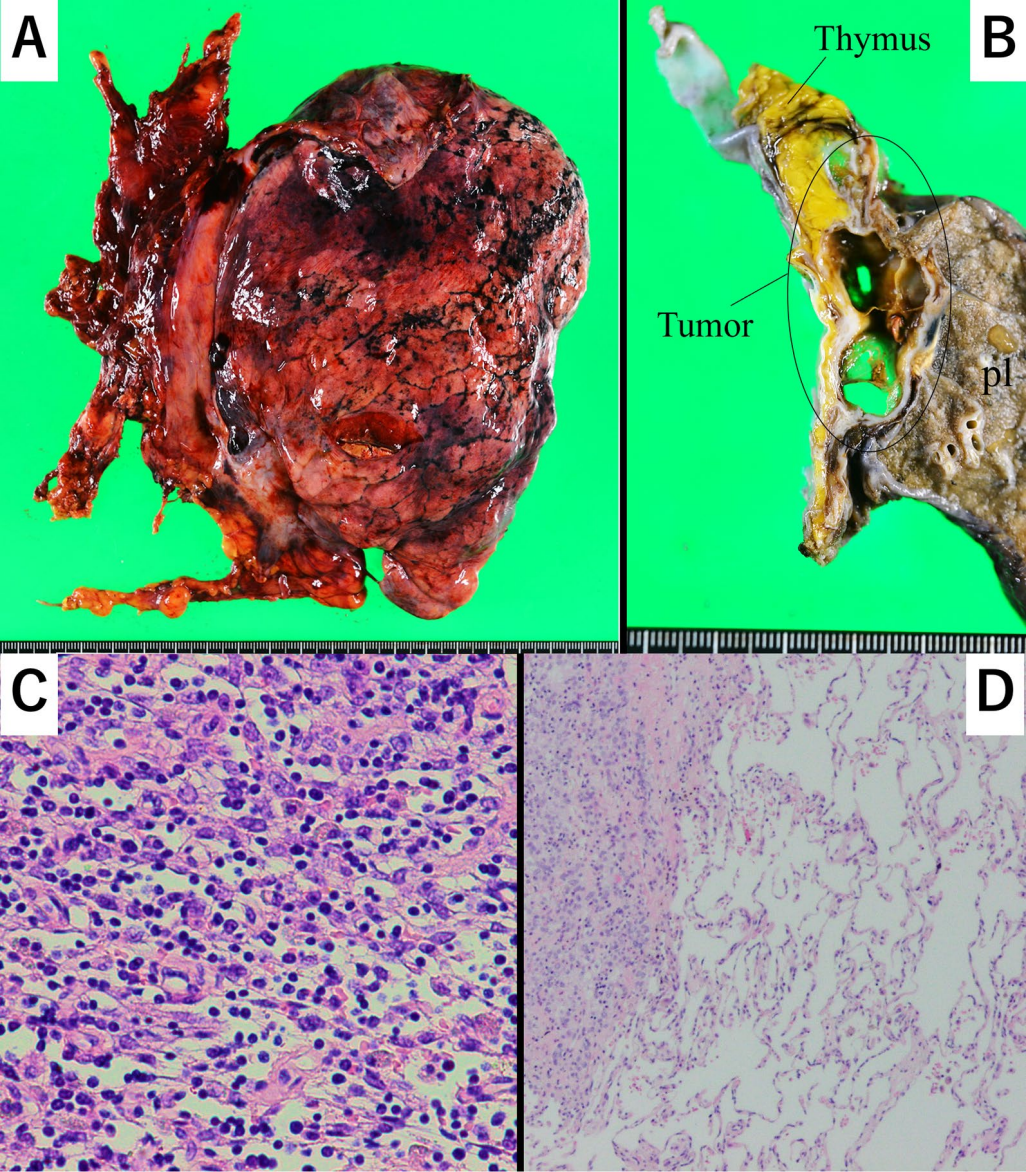
**A** Gross findings of the resected specimen. The thymus, partial parietal, mediastinal, visceral pleura, partial pericardium, and left upper lobe of the lung were resected. The tumor grossly invaded the neighboring structures. **B** Gross findings from the resected specimen after fixation showed tumor shrinkage by induction chemotherapy. **C** Microscopic finding from the resected tumor specimen. The tumor tissue is characterized by a relatively sparse distribution of epithelial cells within a background of abundant lymphoid tissue. Areas with lymphocytes were also observed. The tumor was diagnosed as Type B3 thymoma. **D** Microscopic finding from the resected tumor specimen

The purpose of this surgery was to remove all the tumor sites where thymoma was located before the induction chemotherapy. The surgery included a total thymectomy and partial resections of the parietal pleura, mediastinal pleura, visceral pleura, pericardium, and left upper lobectomy (Video 1). We performed combined resection of a portion of the left phrenic nerve and the left brachiocephalic vein. Complete macroscopic resection was achieved. Postoperative Grade 2 supraventricular tachycardia developed, but the patient was discharged in good condition after 10 days. Pathological analysis confirmed a Type B3 thymoma, ypT3 (lung) N0M0 stage IIIA, with no malignancy in the pericardial or pleural effusions (Fig. 3A–D). Pathologically, the tumor had invaded the lung parenchyma, pericardium, mediastinal pleura, and phrenic nerve. Although no viable tumor cells were detected in the resected visceral pleura, preoperative induction chemotherapy was administered in this case. Therefore, tumor invasion in the resected visceral pleura before chemotherapy was unclear. Intraoperative decisions led to a left upper lobectomy due to invasion of the pulmonary hilum of the left upper lobe near the root of the superior pulmonary vein and the lung parenchyma close to the mediastinal hilum. Pathological examination confirmed the invasion of the lung parenchyma and the superior pulmonary vein at the hilum. The residual viable tumor rate was 50% in evaluating the pathological efficacy of chemotherapy. No recurrence was detected 9 months post-surgery.

## Discussion

We report a case of a large thymoma occupying the left thoracic cavity. The thymoma presented surgical challenges, but was successfully treated with complete resection after preoperative induction chemotherapy. Complete resections of large or advanced thymomas may be difficult, and various treatment strategies have been reported. Multimodality therapy with induction chemotherapy followed by surgical resection is a widely accepted approach [1]. We previously achieved a high rate of complete resection and long-term survival with induction chemotherapy followed by surgery in patients with advanced or recurrent thymoma cases [2, 3]. Based on this experience, we treated the current case using a multidisciplinary approach and achieved a curative resection.

Although no standard chemotherapeutic regimen has been established for thymomas, several regimens have been introduced [3–5]. Yokoi et al. demonstrated that the CAMP chemotherapy regimen for patients with advanced-stage thymomas results in a response rate of 92.9%, 5- and 10-year overall survival rates of 80.7%, and no major adverse events except acceptable neutropenia [3]. Yokoi et al. concluded that CAMP therapy was highly effective for the treatment of advanced-stage thymomas. In the current case, the patient was treated with the CAMP regimen before surgery, demonstrating the feasibility and safety of this multidisciplinary treatment.

No standard surgical procedure for large or advanced-stage thymomas has been established. For curative complete resection in patients with thymomas invading adjacent structures, total thymectomy must be combined with resection of adjacent invaded structures to prevent postoperative local recurrence or pleural dissemination. In the current case, the thymoma was exceptionally large; CT showed the involvement of multiple adjacent organs, including the pericardium, pleura, phrenic nerve, and left upper lobe of the lung. Notably, the tumor extensively abutted the pleura, encompassing the mediastinal, parietal, and visceral pleura; the visceral pleura of the left lower lobe was also involved, raising concerns about pleural dissemination if these areas were not treated. To prevent pleural dissemination, complete resection would have entailed thymectomy with left total pleurectomy or extrapleural pneumonectomy. Considering the patient’s age and comorbidities, we performed total thymectomy, along with partial resections of the parietal, mediastinal and visceral pleura, pericardium, and left upper lobectomy. This approach achieved complete histological resection, mitigating the risk of recurrence.

## Conclusions

Herein, we report a case of giant thymoma. Surgical treatment alone may not have achieved complete resection, but after inducing significant tumor shrinkage with preoperative CAMP therapy, we were able to achieve complete resection. This treatment strategy may be effective in large thymoma cases.

### Supplementary Information

Supplementary Material 1: The surgical procedure aimed to completely remove the entire extent of the tumor existing before chemotherapy. This included a total thymectomy and partial resections of the partial parietal, mediastinal, visceral pleura, pericardium, and left upper lobectomy. The surgical approach involved a left hemi-clamshell incision at the fourth intercostal space. The procedure began with a total thymectomy. Due to tumor invasion into the pericardium, a partial resection of the pericardium was performed. We resected a part of the pericardium anterior to the phrenic nerve, which was in contact with the tumor before chemotherapy, as shown in imaging. The left brachiocephalic vein was dissected, sutured, and resected en bloc. After thymectomy, the left fourth intercostal space was widened, and the pleura (visceral and mediastinal) previously abutted by the tumor before chemotherapy were excised. A portion of the phrenic nerve was resected to obtain surgical margins in the areas of macroscopic tumor invasion. The left main pulmonary artery was taped, and the visceral pleura on the lung side, which was in contact with the tumor before chemotherapy, was partially resected. Subsequently, a left upper lobectomy was performed. The surgical procedure included total thymectomy, left upper lobectomy, and partial resection of the parietal, mediastinal, and visceral pleura and pericardium, which were in contact with the tumor before chemotherapy. The defect in the pericardium resulting from the resection was repaired with a Gore–Tex patch. Initially, the sternum was approached using a hemi-clamshell incision at the fourth intercostal space on the left side. Toward the end of the surgery, access for pericardial reconstruction and partial resection of the visceral pleura of the left lower lung lobe was inadequate. Consequently, a median full sternotomy was performed to complete the procedure. Concerning the hemi-clamshell incision, we planned an L-shaped incision at the fourth intercostal space after determining that lobectomy was necessary due to tumor invasion into the hilum pulmonary parenchyma. The intercostal muscle was incised after thymectomy because access to lobectomy, pericardial reconstruction, and parietal pleurectomy with only a median sternotomy was difficult. We resected a part of the pericardium anterior to the phrenic nerve, which was in contact with the tumor before chemotherapy, as shown in imaging. The total duration of the operation was 341 minutes, and the blood loss was 1,028 ml.

## Data Availability

The data sets supporting the findings and inferences of this case report are included in this article.
